# Nocturia in patients with cognitive dysfunction: a systematic review of the literature

**DOI:** 10.1186/s12877-020-01622-8

**Published:** 2020-07-06

**Authors:** Rebecca Haddad, Thomas F. Monaghan, Charles Joussain, Véronique Phé, Wendy Bower, Saskia Roggeman, Gilberte Robain, Karel Everaert

**Affiliations:** 1 Department of Urology, Ghent University Hospital, Corneel Heymanslaan 10, 9000 Ghent, Belgium; 2 GRC 001 GREEN Neuro-Urology Research Group, Sorbonne Université Rothschild Academic Hospital AP-HP, F-75012 Paris, France; 3 Department of Urology, SUNY Downstate Medical Center, Brooklyn, NY USA; 4 Medical School Paris Île-de-France Ouest Inserm U1179, Versailles Saint-Quentin University, Versailles, France; 5 Department of Physical Medicine and Rehabilitation, Raymond-Poincaré Academic Hospital AP-HP, Garches, France; 6 Department of Urology, Sorbonne Université Pitié-Salpêtrière Academic Hospital AP-HP, Paris, France; 7 Faculty of Medicine, Dentistry & Health Sciences, The University of Melbourne, Melbourne, Australia

**Keywords:** Nocturia, Lower urinary tract symptoms, Cognitive dysfunction, Systematic review, Epidemiology

## Abstract

**Background:**

The objective of this study is to evaluate current literature on the association between cognitive dysfunction and nocturia.

**Methods:**

A systematic review following Preferred Reporting Items for Systematic Reviews and Meta-Analyses (PRISMA) statement was conducted through MEDLINE, EMBASE and COCHRANE databases and completed in November 2019. Randomized and non-randomized studies were included if they assessed the association between cognitive dysfunction and nocturia in older participants with or without neurological diseases. The quality of included studies was evaluated using the Risk of Bias Assessment tool for Non-randomized Studies (RoBANS).

**Results:**

A total of 8 cross-sectional studies conducted in older patient populations met the criteria for inclusion. A statistically significant association was identified in 6 studies on univariate analysis, which persisted in 2 studies after controlling for confounding factors. The association between cognitive dysfunction and nocturia was positive for all 6 significant analyses. The overall risk of bias was unclear.

**Conclusion:**

A significant positive association between cognitive dysfunction and nocturia was identified. However, research has been limited to cross-sectional studies, which precludes identification of causality between cognitive dysfunction and nocturia. Heightened awareness of the complex interplay between cognition and nocturia would allow professionals involved in the care of cognitively impaired patients with concomitant nocturia to more effectively manage these symptoms.

## Background

Nocturia, defined as the act of waking to void during the hours of intended sleep, is among the most common and bothersome lower urinary tract symptoms (LUTS) [1, 2]. Although nocturia is a pervasive complaint across different patient populations, older people are disproportionally affected, with clinically-significant symptoms reported in up to 60% of patients over 70 years of age [3]. Nocturia has been associated with significant morbidity and mortality [4] and shown to have a direct adverse effect on sleep architecture [5, 6]. Among older patient populations, nocturia confers an increased risk of both falls and hip fractures [7, 8], and it stands to reason that this effect may be more pronounced in the setting of concomitant cognitive impairment [9].

Notably, there exists a robust association between cognitive dysfunction - which refers to deficits in attention, verbal and nonverbal learning, short-term and working memory, visual and auditory processing, problem solving, processing speed, motor functioning - and other LUTS, particularly urinary incontinence [10–12]. Moreover, current literature has identified a diverse array of risk factors common to both nocturia and cognitive dysfunction, including aging, comorbidities [13], brain lesions - particularly those affecting the hypothalamic-pituitary axis [14], and sleep disturbances [15]. However, relative to other LUTS, the relationship between nocturia and cognitive dysfunction remains poorly characterized. Increased awareness of the complex interplay between cognition and nocturia would facilitate the evaluation and management of cognitively impaired patients with concomitant nocturia.

The objective of this systematic review was to evaluate available evidence on the association between cognitive dysfunction and nocturia.

## Methods

This systematic review was conducted according to the Preferred Reporting Items for Systematic Reviews and Meta-Analyses (PRISMA) statement [16].

### Eligibility criteria

#### Types of studies

Original studies including randomized controlled trials (RCTs), non-RCTs, single-arm cohort studies, case-control studies, cross-sectional studies, and case series were included. Review articles and meta-analyses were not included, but references from these studies were reviewed, and eligible articles were subsequently retrieved.

#### Population

Only studies involving adults were selected. No limits on gender, ethnicity or setting were applied. The presence of nocturia had to have been reported in participants, regardless of the method of assessment or the threshold used to define nocturia. Cognitive dysfunction had to have been assessed by either a validated neuropsychological test or defined via formal diagnosis of a neurocognitive disorder.

#### Outcomes

The primary outcome was the association between cognitive dysfunction with neurodegenerative disorders of aging and nocturia. Measurement of this association was specified by an effect size indicator and/or a statistical significance level.

### Search strategy and information sources

The MEDLINE, EMBASE, COCHRANE and CENTRAL databases were used with specific keywords (MeSH or Emtree terms) combined with Boolean operators. The complete search strategy is available as supplementary data (Additional File 1). The search was completed in November 2019 and no date limits were applied. No limit of language was applied. References cited in systematic reviews or meta-analyses were searched. Once key articles were identified, additional searches were performed on PubMed using the “Related Articles” search feature.

### Data collection and analysis

#### Study selection

**Fig. 1:**
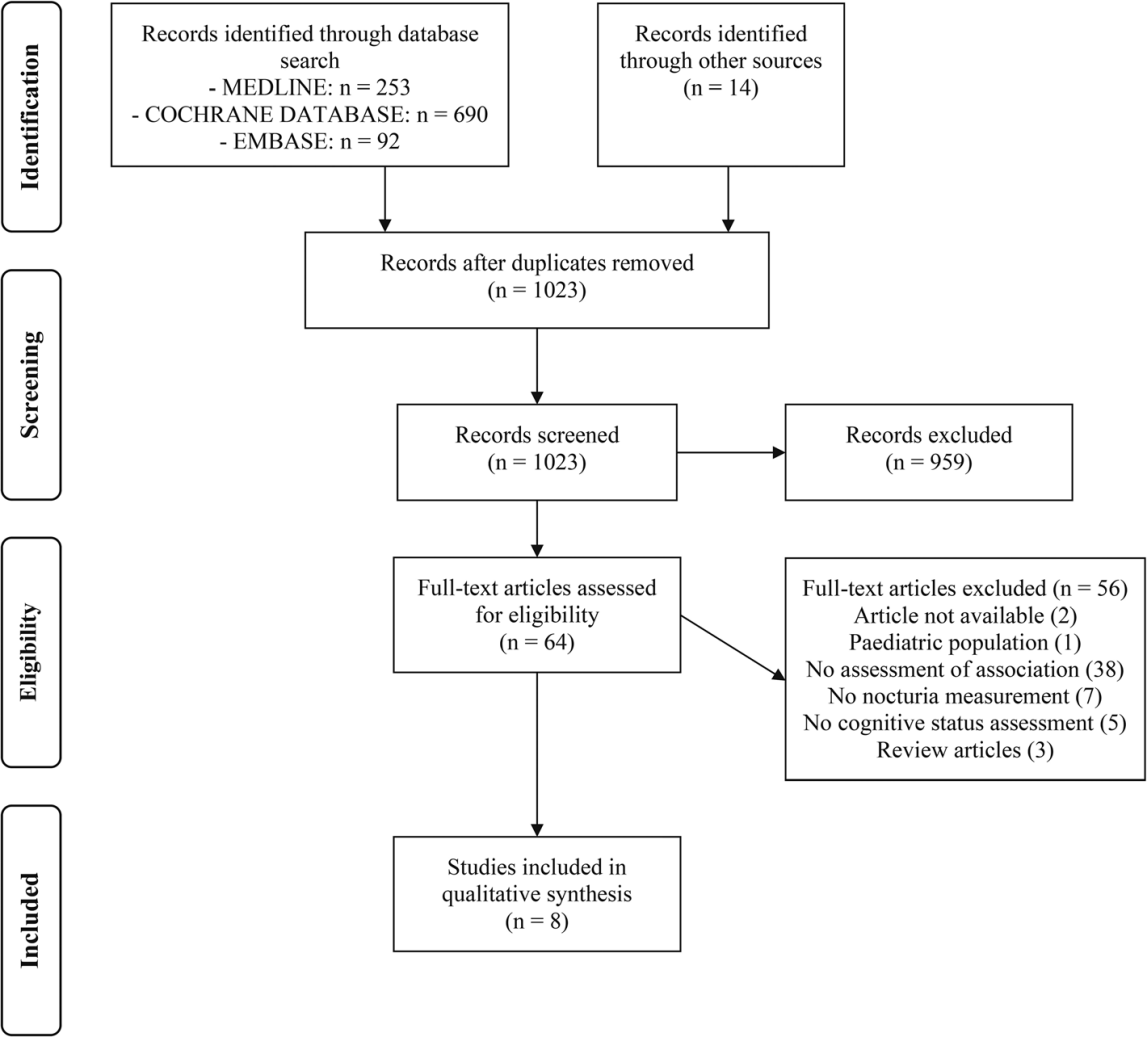
Flow diagram

Two review authors (RH & TM) independently screened the titles and abstracts yielded by the search after inclusion criteria had been applied. Full articles were obtained for all titles that appeared to meet the inclusion criteria or for which there was any uncertainty. Review authors then screened the complete articles and independently determined whether these met the inclusion criteria. Disagreement was resolved through discussion. Rationale for excluding trials was recorded. Figure 1 summarizes this selection process according to the PRISMA statement.

#### Data extraction

Duplicate data were extracted independently (RH & TM) using a standard spreadsheet, which captured information on study design, population, and the association between cognitive dysfunction and nocturia. A summary is provided in the results section (Tables 1 & 2).

**Table 1:** Study characteristics

Study	Population	Nocturia Assessment	Cognitive dysfunction Assessment
Dutoglu et al. (2019) [17]	858 outpatients admitted to a geriatric center Female: 100% Mean (sd) age: 74.1 (8.2) years	“Generally, during the past 30 days, how many times do you usually urinate after you have gone to sleep at night until the time you got up in the morning?” Cut-off: 1, 2, 3, 4 or more Prevalence: 19.0, 24.2, 18.4, and 24.1% respectively	MMSE and Dementia diagnosed using the DSM V Mean (sd) MMSE score: 24.7 (4.9), 25.0 (4.2), 24.9 (3.7), 24.2 (4.4), 23.9 (4.9) in patients with 0, 1, 2, 3 or ≥ 4 nocturia episodes respectively Prevalence (dementia): 4.4%
Jung et al. (2017) [11]	376 patients with probable Alzheimer’s disease Female: 51.1% Age range: 56–92 years	OABSS Mean (sd) number of nocturia episodes: 1.2 (0.8), 1.2 (0.9) and 1.6 (1.0) in patients with OABSS ≤5, 6–11 or ≥ 12 respectively Prevalence: NR	MMSE and CDR scale Mean (sd) MMSE score: 14.4 (7.6) in patients with OAB Mean (sd) CDR score: 2.3 (0.9) in patients with OAB
Zhang et al. (2016) [18]	454 patients with Parkinson’s disease Female: 42.7% Mean (sd) age: 61.5 (10.9) years	NMSS Mean (sd) NMSS score for nocturia: 2.4 (3.3) Cut-off: NR Prevalence: 47.2%	MoCA Mean (sd) MoCA score: 23.7 (4.5) Prevalence (MoCA ≤25): 58.1%
Scullin et al. (2013) [19]	143 patients with Parkinson’s disease Female: 35% Mean (sd) age: 64.7 (9.0) years	“When you awaken during the night, how often do you urinate?” on sleep questionnaire drawn from existing studies; Nocturia frequency evaluated on 4-point Likert scale (1 = “never,” 4 = “very often”) Prevalence: NR	Impulsivity determined by at least 1 “yes” to the Minnesota Impulse Disorder Interview (MIDI) questions Prevalence: 26.6%
Vaughan et al. (2013) [20]	63 patients with Parkinson’s disease Female: 35% Mean (sd) age: 63 (9.7) years	IPSS Cut-off: ≥2 voids/nights Prevalence: 61%	MMSE Mean (sd) MMSE score: 28.6 (1.5) in patients without nocturia and 28.5 (1.9) in patients without Prevalence: NR
Galizia et al. (2012) [21]	1288 community-dwelling individuals Female: 57% Mean (sd) age: 74.2 (6.3) years	History taking Cut-off: ≥2 voids/nights Prevalence: 45.8%	MMSE Mean (sd) MMSE score: 25.3 (4.8) Prevalence: NR
Lee et al. (2012) [22]	299 community-dwelling men Mean (sd) age: 71.2 (5.0) years	History taking Cut-off: ≥2 voids/nights Prevalence: 56.0%	MMSE Mean (sd) MMSE score: 25.6 (3.4) Prevalence: NR
Burgio et al. (2010) [23]	1000 Medicare beneficiaries Female: 50% Mean (sd) age: 73.8 (NR) years	History taking Cut-off: ≥2 voids/nights Prevalence: 58.5%	MMSE Mean (sd) MMSE score: 25 (4.9) Prevalence (MMSE < 24): 29.8%

*BPH* Benign prostatic hypertrophy, *CI* Confidence interval, *CDR* Clinical Dementia Rating; *DSM* Diagnostic and Statistical Manual of Mental Disorders, *HAMA* Hamilton Anxiety Rating Scale, *HAMD* Hamilton Depression Rating Scale, *IPSS* International Prostate Symptom Score, *MoCA* Montreal Cognitive Assessment, *MMSE* Mini-Mental State Examination, *NMSS* Non-Motor Symptom Scale, *NR* Not reported, *OABSS* Overactive Bladder Symptom Score, *OR* Odds ratio, *r* Correlation coefficient, *sd* Standard deviation, *UPDRS* Unified Parkinson’s Disease Rating Scale

**Table 2:** Association between cognitive dysfunction and nocturia

Study	Univariate analysis	Multivariable analysis
Dutoglu et al. (2019) [17]	Lower MMSE scores in patients with ≥2 nocturia episodes compared to those with < 2 episodes; MCID observed only for the group with at least 4 nocturnal voids compared to the group with 1 nocturnal void No difference in dementia prevalence	Not performed
Jung et al. (2017) [11]	No significant correlation between nocturia and MMSE Significant correlation between nocturia and CDR scale: r = 0.23; MCID: not assessable	Not performed
Zhang et al. (2016) [18]	Significant difference of nocturia prevalence in patients with cognitive dysfunction vs. without 56.3% vs. 36.8%. Mean (sd) NMSS nocturia sub-score significantly higher in patients with cognitive dysfunction vs. without 2.9 (3.4) vs. 1.7 (3.0); MCID: not assessable	Urinary disorders (including nocturia) as a significant risk factor for cognitive dysfunction^a^: OR 1.7, 95% CI [1.1–2.8]
Scullin et al. (2013) [19]	No significant difference in Mean (sd) nocturia frequency score in patients with impulsivity vs. without impulsivity	-
Vaughan et al. (2013) [20]	No significant difference in MMSE score in patients with nocturia vs. without nocturia	-
Galizia et al. (2012) [21]	Mean (sd) MMSE score significantly lower in subjects with vs. without nocturia in univariate analysis: 25.0 (5.2) vs. 25.6 (4.4); MCID not reached	Not performed
Lee et al. (2012) [22]	Mean (sd) MMSE score significantly lower in subjects with vs. without nocturia in univariate analysis: 24.4(4.0) vs. 25.9(3.4); MCID reached	Higher MMSE protective factor of nocturia^b^: OR 0.6 95%CI [0.5–0.9]
Burgio et al. (2010) [23]	MMSE protective factor of nocturia with OR 0.9 (CI non available) MCID not assessable	Non-significant association^c,d^

*BPH* Benign prostatic hypertrophy; *CI* Confidence interval, *CDR* Clinical dementia rating scale (MCID: 1–2 point increase indicative of a meaningful decline), *HAMA* Hamilton Anxiety Rating Scale, *HAMD* Hamilton Depression Rating Scale, *MCID* Minimal clinically important difference, *MMSE* Mini-Mental State Examination (MCID: 1–3 point decrease indicative of a meaningful decline), *NMSS* Non-Motor Symptom Scale (MCID: 13.91 point increase indicative of a meaningful change), *OR* Odds ratio, *r* Correlation coefficient, *sd* standard deviation, *UPDRS* Unified Parkinson’s Disease Rating Scale

Variables included in multivariable analysis:

^a^Age, age of onset, gender, education level, scores of speech, facial expression, tremor, rigidity, bradykinesia and axial impairment in the UPDRS, total HAMD and HAMA scores, presence of sleep/ fatigue, perceptual problems/hallucinations, attention/memory, gastrointestinal domains from NMSS

^b^History of BPH, age, education, depression, alpha-blocker, transitional zone volume of prostate

^c^Age, ethnicity, obesity, urban status (vs rural)

^d^Age, ethnicity, hypertension, lower limb oedema, history of urinary incontinence, urban status (vs rural)

#### Assessment of the risk of bias in individual studies

**Fig. 2:**
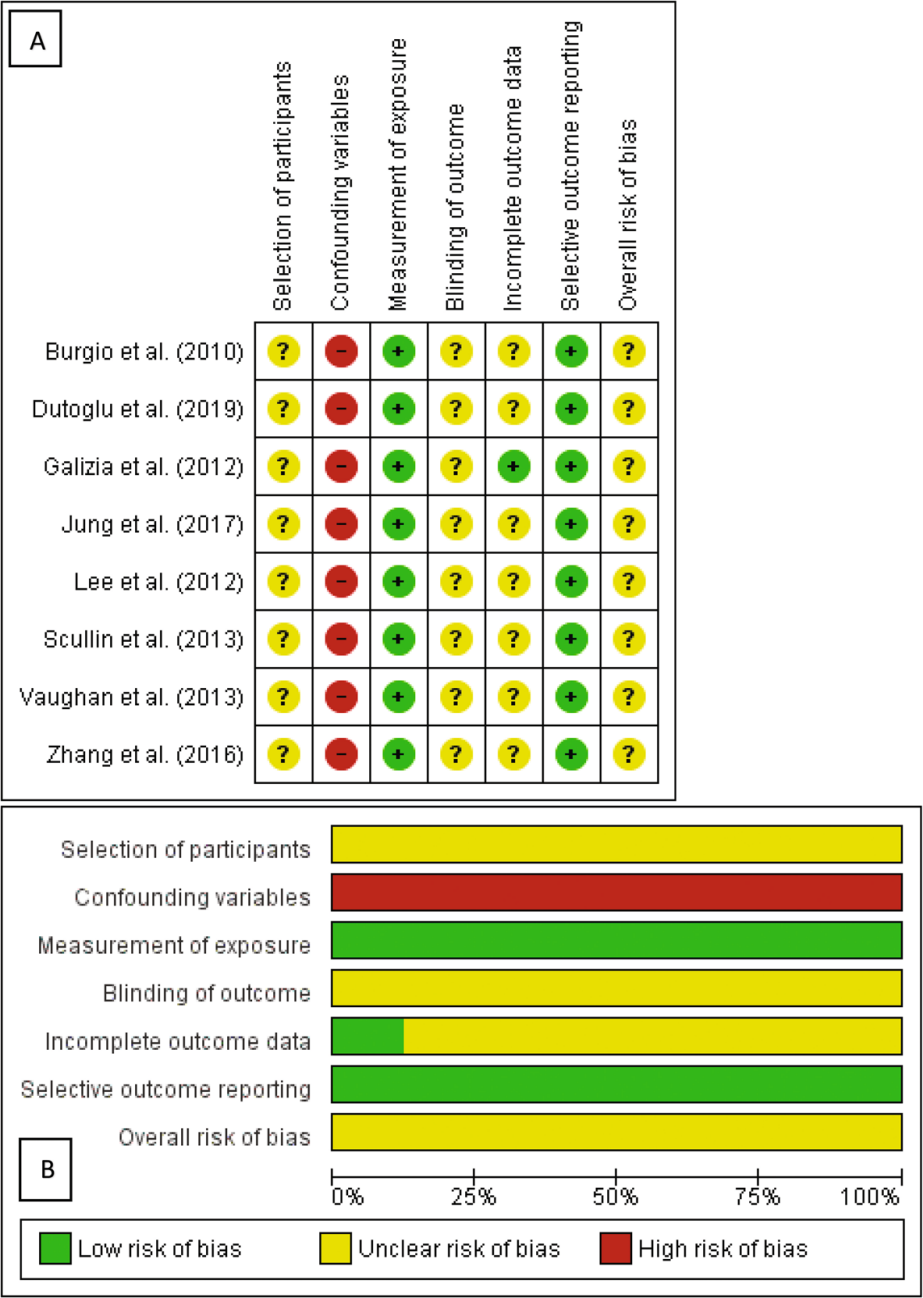
Risk of bias in included studies. 2A. Risk of bias summary: review authors’ judgements about each risk of bias item for each included study. 2B. Risk of bias graph: review authors’ judgements about each risk of bias item presented as percentages across all included studies.?: unclear risk of bias; −: high risk of bias; +: low risk of bias

The quality of non-randomized studies was assessed using the validated Risk of Bias Assessment tool for Non-randomized Studies (RoBANS) [24]. Dispositions were made independently by two authors (RH & TM). Disagreements were resolved first by discussion, and then by consulting a third author for arbitration as warranted (KE). A summary is provided in the results section (Fig. 2).

## Results

### Study selection

A flowchart of the database search and selection process is provided in Fig. 1. Of the 1023 non-duplicate articles identified from the databases, 64 articles were eligible for systematic review. A total of 8 studies were included in the final analysis.

### Study characteristics

Study characteristics are summarized in Table 1. The 8 studies included all featured a cross-sectional study design and community-dwelling participants. Four studies specifically enrolled older patients [17, 21–23], 3 studies [18–20] were conducted in Parkinson’s disease (PD) patients and 1 enrolled participants with probable Alzheimer’s disease (AD) [11]. Cognitive dysfunction was assessed by Mini-Mental State Examination (MMSE) in 6 studies [11, 17, 20–23]**,** and also the Clinical Dementia Rating (CDR) scale in 1of these studies [11]. The Montreal Cognitive Assessment was used in 1 study [18], and a specific validated scale for impulsivity in PD in 1 study [19]. The prevalence of cognitive dysfunction ranged from 4 to 58% in the studies which reported this parameter [17, 18, 23]. Nocturia was defined as self-reported awakening from sleep to pass urine 2 or more times per night [21–23], or without any definite threshold [11, 17] or by symptom questionnaires [18–20]. Overall, nocturia was reported in 19 to 61% of study participants [17, 20].

### Assessment of the association between cognitive dysfunction and nocturia

The association between cognitive dysfunction and nocturia was statistically significant in 6 studies [11, 17, 18, 21–23], all in the same direction (Table 2). In 1 study [18], nocturia was more frequently identified in PD patients with cognitive dysfunction compared to those without (56% vs. 37% respectively). In 4 other studies [17, 21–23], a higher MMSE score was a protective factor for nocturia, whereas Jung et al. (2017) did not find any correlation between MMSE score and nocturia in patients with probable AD. Lee et al. (2012) determined that a higher MMSE score was an independent protective factor after adjustment for multiple potential confounders (OR 0.6, 95% CI [0.5–0.9], *p* = 0.006). In the study by Zhang et al. (2016), this association was independent in multivariable analysis (OR 1.7, 95%CI [1.1–2.8], *p* = 0.03), but it should be noted that the group studied the entire urinary domain (including nocturia) of the Non-Motor Symptoms Scale (opposed to nocturia as a standalone entity). Minimal clinically important difference in cognitive scores was reached in two of the three studies where assessment was possible [17, 22].

### Risk of bias in included studies

The risk of biases is summarized in Fig. 2. Regarding the selection of participants, the risk of bias was unclear in all studies, since no criteria could be applied to cross sectional studies using the RoBANS instrument. Nevertheless, 3 studies [21–23] randomly selected their participants, with a response rate ranging from 75 to 98%. Of the 8 studies included, only 3 controlled their analyses for confounding factors [18, 22, 23]. Potential confounding factors considered included age (in all of these studies), ethnicity [23], gender [18], education (in all of these studies), PD history (age of onset, disease severity assessed by the Unified PD Rating Scale, hallucination or gastrointestinal symptoms) in the study by Zhang et al. (2016), urological history (transitional volume zone, history of benign prostate hyperplasia, alpha blockers intake) in the study by Lee et al. (2012) and comorbidities such as sleep or fatigue symptoms [18], lower limbs edema, hypertension, obesity or urinary incontinence [23], depression [18, 22] and anxiety [18]. When gender was not considered, analyses were stratified according to it. All but 1 of the studies [23] were deemed to be at low risk for bias in the measurement of exposure. Except for one study [21], no studies published information on blinding of outcome assessments or incomplete data. Although no studies referenced a published protocol with pre-defined outcomes, expected outcomes were reported in the results section of all 8 studies.

## Discussion

### Summary of evidence

To date, only 8 cross-sectional studies involving older populations, patients with PD, or patients with AD have assessed the association between cognitive dysfunction and nocturia. The overall risk of bias of these studies was unclear. All 6 studies that identified a statistically significant association between nocturnal voiding frequency and cognitive dysfunction did so in the same direction [11, 17, 18, 21–23]. This association was shown to be independent in 2 studies. Accordingly, current literature suggests that a lower cognitive function is associated with a higher risk of nocturia, although more research is indeed needed to further elucidate the relationship between cognitive dysfunction and nocturia.

Although several studies have found a significant association between nocturia and cognitive dysfunction, the clinical relevance of these associations must be questioned.

### Strengths and weaknesses

This systematic review is the first on the topic of cognitive dysfunction and nocturia. We identified studies which included nearly 4500 older patients in total.

**Fig. 3:**
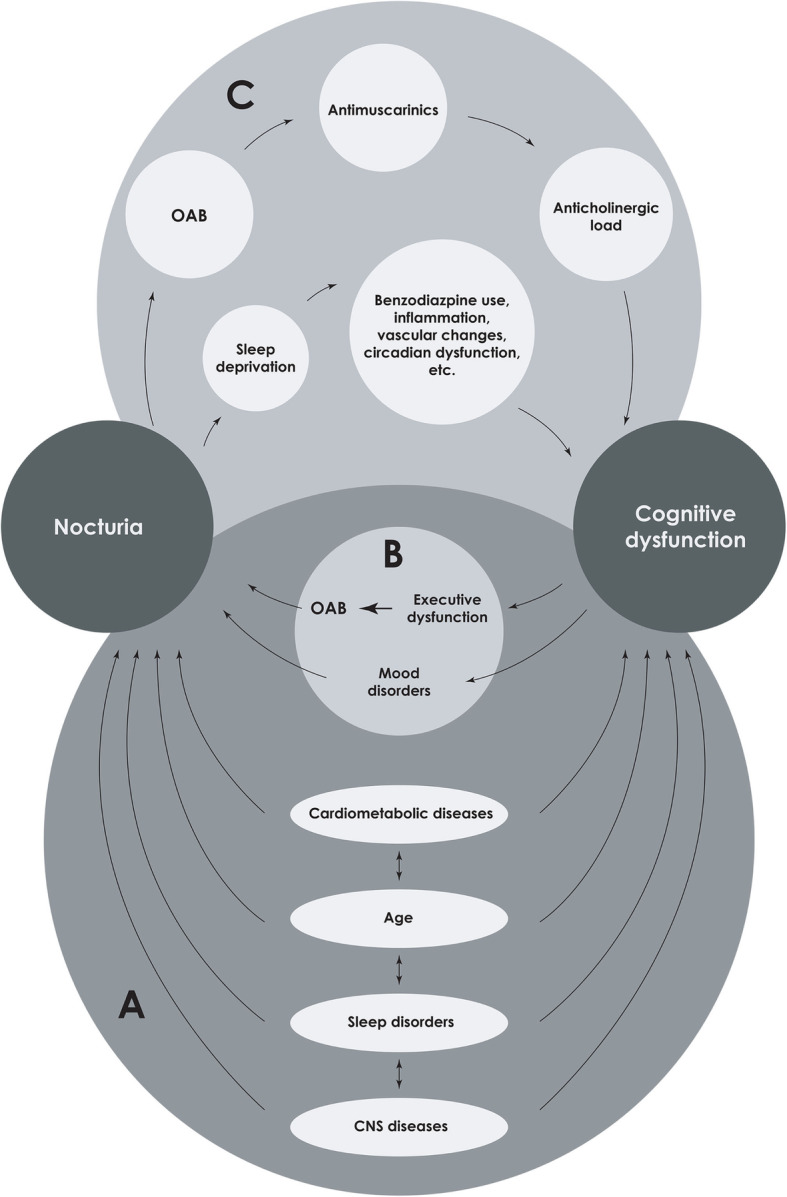
Hypotheses for the association between cognitive dysfunction and nocturia. CNS: central nervous system; OAB: overactive bladder.

However, the included study populations were heterogeneous, and varied in the means by which potential confounding factors were assessed. Indeed, age, sleep disorders, cardio-metabolic and central nervous system diseases are the main confounding factors in the relationship between cognitive disorders and nocturia (Fig. 3). If age is considered in each study including a multivariable analysis, only Zhang et al. (2016) have considered sleep disorders, cardio-metabolic diseases were partially considered in the study by Burgio et al. (2010) and neurological diseases were ignored if we exclude the study in PD patients [18]. Moreover, the definition and assessment of nocturia (e.g., self-reported voiding frequency vs. symptom questionnaire data) varied across studies, which was consistent with a previous study [25]. At the time of publication, none of the studies included in this review used the exact terminology recommended by the International Continence Society, which states that each void is preceded and followed by sleep or the intention to sleep [2, 26]. Nevertheless, several studies used the clinically significant threshold of two or more nocturnal voids [17, 20–23]. In addition, none of the studies assessed nocturia on frequency volume charts, but only on validated questionnaires or by history taking. This may have led to a risk of misclassification bias. Indeed, it has been shown that nocturia prevalence is overestimated when using questionnaires [27, 28]. Mis-estimating (over- or under-estimating) nocturnal voiding frequency to a greater extent in patients with cognitive dysfunction compared to those without could lead to this bias. Regarding cognitive dysfunction, the MMSE, was used in most of the included studies. It is a short, patient-friendly instrument, but may be influenced by educational attainment and age. These limitations, coupled with the relatively small number of studies which met the criteria for inclusion, precluded quantitative synthesis of study results.

Although several studies have found a significant association between nocturia and cognitive dysfunction, the clinical relevance of these associations must be questioned. While it is difficult to estimate for some studies, it appears to have been observed in others. For example, in the study by Lee et al. (2012), the observed difference in MMSE between patients with nocturia and those without nocturia was 1.5 points, which is considered clinically significant [29].

### Explanatory hypotheses

The fact that only cross-sectional studies met the criteria for inclusion in this systematic review precludes identification of the precise cause-and-effect relationship between cognitive dysfunction and nocturia. Nevertheless, several non-mutually exclusive hypotheses may explain this association (Fig. 3).

Cognitive dysfunction and nocturia share many of the same risk factors (Part (a) of Fig. 3) including central nervous system pathologies [30], altered circadian rhythms of key hormones [15, 31, 32], reduction in sex hormones [33, 34], sleep disorders [15, 34], comorbidities [13, 34], polypharmacy and modifiable lifestyle factors [13, 34, 35]. Furthermore, cognitive dysfunction could give rise to nocturia (part (b) of Fig. 3), mainly through impaired executive function, which is associated with overactive bladder syndrome [11, 12], and decrease in physical activity [36]. These two manifestations are known causes of nocturia [3, 37]. Conversely, nocturia could give rise to cognitive impairment (part (c) of Fig. 3). Antimuscarinic agents use, a first-line therapy in the management of overactive bladder, may contribute to cognitive dysfunction [38] through an increase of the anticholinergic load. Moreover, nocturia-induced sleep impairment might lead to cognitive decline [13], via factors such as benzodiazepine use [15].

## Conclusions

Nocturia is a complex and multifactorial condition, which may be the result of a primary abnormality of the genitourinary tract or a symptom of an underlying medical condition. The current framework for evaluation and management of nocturia broadly divides nocturia into four distinct etiologies: excessive nocturnal urine production (“nocturnal polyuria”), excessive 24-h urine production (“global polyuria”), reduced bladder capacity (functional or extrinsic), and sleep disorders (primary or secondary). However, the cause-and-effect relationship between nocturia and cognitive dysfunction remains poorly understood, and this may fall into overlapping pathophysiologic phenotypes. Nevertheless, the findings from this review suggests that nocturia is associated with cognitive impairment, and this could have consequential impacts on clinical practice. Indeed, it could make it possible to further appropriate assessment of signs or symptoms of memory loss in patients with nocturia, or to systematically look for this bothersome symptom in patients with cognitive dysfunction.

## Supplementary information

**Additional file 1.** Electronic search strategy. Description of the electronic search strategy used to find articles on the different databases.

## Data Availability

Data sharing is not applicable to this article as no datasets were generated or analysed during the current study.

## Abbreviations

AD: Alzheimer’s disease
CDR: Clinical Dementia Rating
LUTS: Lower urinary tract symptoms
MMSE: Mini-Mental State Examination
PD: Parkinson’s disease
PRISMA: Preferred Reporting Items for Systematic Reviews and Meta-Analyses
RCTs: Randomized controlled trials
RoBANS: Risk of Bias Assessment tool for Non-randomized Studies
