# Supplementary material for: Nocturia in patients with cognitive dysfunction: a systematic review of the literature
Source: BMC Geriatr. 2020 Jul 6;20:230. doi: 10.1186/s12877-020-01622-8 (PMC7336631; doi:10.1186/s12877-020-01622-8)
Supplement: Supplementary file 1 — Additional file 1. Electronic search strategy. Description of the electronic search strategy used to find articles on the different databases. [file 12877_2020_1622_MOESM1_ESM.docx]

**Additional file 1. Electronic search strategy**

### PubMed search strategy

(("nocturia"[MeSH Terms] OR "nocturia"[All Fields]) OR (nocturnal[All Fields] AND ("polyuria"[MeSH Terms] OR "polyuria"[All Fields]))) AND (("neurocognitive disorders"[MeSH Terms] OR ("neurocognitive"[All Fields] AND "disorders"[All Fields]) OR "neurocognitive disorders"[All Fields]) OR ("cognitive dysfunction"[MeSH Terms] OR ("cognitive"[All Fields] AND "dysfunction"[All Fields]) OR "cognitive dysfunction"[All Fields] OR ("cognitive"[All Fields] AND "impairment"[All Fields]) OR "cognitive impairment"[All Fields]) OR ("cognitive dysfunction"[MeSH Terms] OR ("cognitive"[All Fields] AND "dysfunction"[All Fields]) OR "cognitive dysfunction"[All Fields]) OR ("dementia"[MeSH Terms] OR "dementia"[All Fields]) OR MCI[All Fields] OR ("cognitive dysfunction"[MeSH Terms] OR ("cognitive"[All Fields] AND "dysfunction"[All Fields]) OR "cognitive dysfunction"[All Fields] OR ("cognitive"[All Fields] AND "decline"[All Fields]) OR "cognitive decline"[All Fields]) OR ("cognition"[MeSH Terms] OR "cognition"[All Fields] OR ("cognitive"[All Fields] AND "function"[All Fields]) OR "cognitive function"[All Fields]) OR ("cognition disorders"[MeSH Terms] OR ("cognition"[All Fields] AND "disorders"[All Fields]) OR "cognition disorders"[All Fields] OR ("cognitive"[All Fields] AND "disorder"[All Fields]) OR "cognitive disorder"[All Fields]) OR ("cognition disorders"[MeSH Terms] OR ("cognition"[All Fields] AND "disorders"[All Fields]) OR "cognition disorders"[All Fields]) OR ("neuropsychological tests"[MeSH Terms] OR ("neuropsychological"[All Fields] AND "tests"[All Fields]) OR "neuropsychological tests"[All Fields]) OR (("Cogn Int Conf Adv Cogn Technol Appl"[Journal] OR "cognitive"[All Fields]) AND performance[All Fields]) OR ("executive function"[MeSH Terms] OR ("executive"[All Fields] AND "function"[All Fields]) OR "executive function"[All Fields]) OR ("Mem Cognit"[Journal] OR ("memory"[All Fields] AND "cognition"[All Fields]) OR "memory cognition"[All Fields]) OR (mental[All Fields] AND status[All Fields]) OR (("dementia"[MeSH Terms] OR "dementia"[All Fields]) AND tests[All Fields]) OR ("lewy body disease"[MeSH Terms] OR ("lewy"[All Fields] AND "body"[All Fields] AND "disease"[All Fields]) OR "lewy body disease"[All Fields]) OR ("alzheimer disease"[MeSH Terms] OR ("alzheimer"[All Fields] AND "disease"[All Fields]) OR "alzheimer disease"[All Fields]) OR ("frontotemporal dementia"[MeSH Terms] OR ("frontotemporal"[All Fields] AND "dementia"[All Fields]) OR "frontotemporal dementia"[All Fields]) OR ("dementia, multi-infarct"[MeSH Terms] OR ("dementia"[All Fields] AND "multi-infarct"[All Fields]) OR "multi-infarct dementia"[All Fields] OR ("dementia"[All Fields] AND "multi"[All Fields] AND "infarct"[All Fields]) OR "dementia, multi infarct"[All Fields]) OR (Dementia-Parkinsonism[All Fields] AND Non-Alzheimer[All Fields] AND ("plaque, amyloid"[MeSH Terms] OR ("plaque"[All Fields] AND "amyloid"[All Fields]) OR "amyloid plaque"[All Fields] OR ("amyloid"[All Fields] AND "plaques"[All Fields]) OR "amyloid plaques"[All Fields])) OR ("multiple system atrophy"[MeSH Terms] OR ("multiple"[All Fields] AND "system"[All Fields] AND "atrophy"[All Fields]) OR "multiple system atrophy"[All Fields]) OR (corticobasal[All Fields] AND degeneration[All Fields]) OR ("stroke"[MeSH Terms] OR "stroke"[All Fields]) OR ("multiple sclerosis"[MeSH Terms] OR ("multiple"[All Fields] AND "sclerosis"[All Fields]) OR "multiple sclerosis"[All Fields]))

### Cochrane Database search strategy

#1 (nocturia) in Cochrane Reviews, Trials, Special collections

#2 (nocturnal polyuria) in Cochrane Reviews, Trials, Special collections

#3 MeSH descriptor: [Mental Status and Dementia Tests] explode all trees

#4 MeSH descriptor: [Cognitive Dysfunction] explode all trees

#5 MeSH descriptor: [Cognition Disorders] explode all trees

#6 MeSH descriptor: [Neurocognitive Disorders] this term only

#7 MeSH descriptor: [Dementia] explode all trees

#8 MeSH descriptor: [Basal Ganglia Diseases] explode all trees

#9 MeSH descriptor: [Multiple Sclerosis] explode all trees

#10 MeSH descriptor: [Cerebrovascular Disorders] explode all trees

#11 (#1 OR #2) AND (#3 OR #4 OR #5 OR # 6 OR # 7 OR #8 OR #9 OR #10) in Cochrane Reviews, Trials, Special collections

### EMBASE search strategy

('nocturia'/exp OR 'nocturia' OR 'nocturnal polyuria'/exp OR 'nocturnal polyuria' OR (night* NEAR/2 frequency) OR ('night time*' NEAR/2 frequency) OR (night* NEAR/2 urination) OR ('night time*' NEAR/2 urination)) AND ('disorders of higher cerebral function'/exp OR 'disorders of higher cerebral function' OR 'cognitive defect'/exp OR 'cognitive defect' OR 'cognitive impairment no dementia'/exp OR 'cognitive impairment no dementia' OR 'dementia'/exp OR 'dementia' OR 'cognitive decline'/exp OR 'cognitive decline' OR 'neuropsychological test'/exp OR 'neuropsychological test' OR 'executive function'/exp OR 'executive function' OR 'executive function test'/exp OR 'executive function test' OR 'memory'/exp OR 'memory' OR 'memory disorder'/exp OR 'memory disorder' OR 'diffuse lewy body disease'/exp OR 'diffuse lewy body disease' OR 'alzheimer disease'/exp OR 'alzheimer disease' OR 'frontotemporal dementia'/exp OR 'frontotemporal dementia' OR 'multiinfarct dementia'/exp OR 'multiinfarct dementia' OR 'parkinson disease'/exp OR 'parkinson disease' OR 'shy drager syndrome'/exp OR 'shy drager syndrome' OR 'cerebrovascular accident'/exp OR 'cerebrovascular accident' OR 'multiple sclerosis'/exp OR 'multiple sclerosis') AND ([article]/lim OR [article in press]/lim OR [review]/lim) AND ([adult]/lim OR [middle aged]/lim OR [aged]/lim OR [very elderly]/lim) AND [humans]/lim AND [abstracts]/lim AND [embase]/lim) NOT ([embase]/lim AND [medline]/lim)
